# The Role of Categorical Perception and Acoustic Details in the Processing of Mandarin Tonal Alternations in Contexts: An Eye-Tracking Study

**DOI:** 10.3389/fpsyg.2021.756921

**Published:** 2022-02-07

**Authors:** Jung-Yueh Tu, Yu-Fu Chien

**Affiliations:** ^1^PhD/MA Program in Teaching Chinese as a Second Language, National Chengchi University, Taipei, Taiwan; ^2^Department of Chinese Language and Literature, Fudan University, Shanghai, China

**Keywords:** tone sandhi, Mandarin Chinese, tonal alternations, neutralization, eye-tracking

## Abstract

This study investigated the perception of Mandarin tonal alternations in disyllabic words. In Mandarin, a low-dipping Tone3 is converted to a high-rising Tone2 when followed by another Tone3, known as third tone sandhi. Although previous studies showed statistically significant differences in F0 between a high-rising Sandhi-Tone3 (T3) and a Tone2, native Mandarin listeners failed to correctly categorize these two tones in perception tasks. The current study utilized the visual-world paradigm in eye-tracking to further examine whether acoustic details in lexical tone aid lexical access in Mandarin. Results showed that Mandarin listeners tend to process Tone2 as Tone2 whereas they tend to first process Sandhi-T3 as both Tone3 and Tone2, then later detect the acoustic differences between the two tones revealed by the sandhi context, and finally activate the target word during lexical access. The eye-tracking results suggest that subtle acoustic details of F0 may facilitate lexical access in automatic fashion in a tone language.

## Introduction

Mandarin Chinese is a tonal language, which uses pitch to distinguish lexical meaning. It has four lexical tones, a high level Tone1, a mid-rising Tone2, a low-dipping Tone3, a high-falling Tone4 as well as a neutral tone (Chao, 1930). With this tonal inventory, Mandarin is well-known for its third tone sandhi, where a low dipping Tone3 (T3) immediately followed by another T3 is altered into a rising tone, similar to the mid-rising Tone2 (T2) (Chao, 1930; Lin, 2007). This third tone sandhi leads to the situation that the sandhi-rising (SR) T3 and canonical-rising (CR) T2 are both realized as rising tones and they seem to be neutralized in the given context. Neutralization is a phenomenon in which two different phonemes are realized as the same sound in certain phonetic environments. The third tone sandhi rule is traditionally/pedagogically described as a T3 becoming a T2 when followed by another T3. The extent of neutralization between sandhi-rising T3 (SR-T3) and canonical-rising T2 (CR-T2), however, still remains a controversial issue. Previous studies comparing SR-T3 and CR-T2 have suggested incomplete neutralization in acoustic details (Peng, 2000; Zhang and Lai, 2010; Yuan and Chen, 2014) but complete neutralization in perception in identification tasks (Wang and Li, 1967; Peng, 2000). In other words, although previous studies showed statistically significant differences in F0 between a SR-T3 and a CR-T2, native Mandarin listeners failed to correctly categorize these two tones in perception tasks (Peng, 2000).

With the development of research methodology, the perception and processing of Mandarin tones have been explored through eye-tracking and Electroencephalography (EEG) technology. In the field of phonetics, eye-tracking experiments were initially conducted to investigate the perception of segmental sounds. For example, the eye-tracking study of consonants by McMurray et al. (2002, 2009) found that participants can perceive within-category voice onset time (VOT) differences of 5 ms. Their findings demonstrated effects of word initial VOT on lexical access, and also support models of spoken word recognition in which sub-phonemic detail is preserved in patterns of lexical activation for competing lexical candidates throughout the processing system. Then, eye-tracking techniques were used to explore the perception of Chinese Mandarin tones. Malins and Joanisse (2010) used this method to examine how segmental and tonal information affect Chinese word recognition. Their results showed that in the process of Chinese word recognition, participants integrate segmental and tonal information in a parallel way. Such findings cannot be observed from the results obtained in previous off-line end-state experiments, while the employment of eye-tracking technology can provide more evidence of on-line real-time data to explore language processing. Eye-tracking technique can provide evidence of use of fine-grained acoustic information that is not found in off-line measurements or tasks. It can shed light on the spoken word recognition process, saying how this information modulates target and competitor word activation as the speech signal unfolds. Later, Shen et al. (2013) conducted an eye-tracking experiment on the perception of Mandarin monosyllabic words with T2 and T3, which investigated how lexical tone perception of Mandarin T2 and T3 was influenced by the pitch height of the tone at onset, turning point, and offset. It has found that native Mandarin listeners perceived the tone with high-offset pitch as T2 while they perceived the tone with low-offset pitch as T3. Shen et al. (2013) further explained that a low turning point pitch served as a pivotal cue for T3, and prompted more eye fixations on T3 items, until the offset pitch directed significantly more fixations to the final tone choice. The findings indicated that in the perception of tones, the pitch height at critical points serves as an important perceptual cue. The results support the perspective that perception of tones is an incremental process.

In addition, Qin et al. (2019) compared the processing of Mandarin T1 and T2 by native Mandarin listeners and English listeners learning Chinese as a second language. They conducted an eye-tracking experiment using the visual world paradigm. Based on the phonetic distance between the target tone and the competitor, stimuli were manipulated such that the target tones were categorized into three conditions, including Standard condition (i.e., the target tone was canonical), Close condition (i.e., the target was phonetically closer to the competitor), and Distant condition (i.e., the target was phonetically more distant from the competitor). They found that within-category tonal information influenced both native and non-native participants’ word recognition, but did so in a different way for the two groups. In comparison with the Standard condition, Mandarin participants’ target-over-competitor word activation was enhanced in the Distant condition and inhibited in the Close condition, while English participants’ target-over-competitor word activation was inhibited in both the Distant and Close conditions.

Meanwhile, the processing and representation of Mandarin disyllabic words are relatively understudied and need more research. Since Mandarin T3 sandhi involves in contexts, it may provide more information to examine T3 sandhi not only in isolation but also in contexts. Chien et al. (2016) conducted an auditory–auditory priming lexical decision experiment to investigate the processing of Mandarin third tone sandhi words during spoken word recognition and their mental representations. In their priming experiment, each disyllabic tone sandhi target word (e.g., /tʂ*^h^*u3 li3/) was preceded by one of three monosyllabic primes: a T2 prime (Surface-Tone overlap, /tʂ*^h^*u2/), a T3 prime (Underlying-Tone overlap, /tʂ*^h^*u3/), or a control prime (Baseline condition, /tʂ*^h^*u1/). Their results showed that T3 primes (Underlying-Tone) elicited significantly stronger facilitation effects for the sandhi targets than Tone 2 primes (Surface-Tone), with little effect of target frequency on the pattern of the priming effects. Thus, they proposed that Mandarin third tone sandhi words are represented as /T3 T3/ in the mental lexicon.

The EEG technique has also been applied to research on the perception and processing of Mandarin disyllabic words. For instance, Chien et al. (2020) used the oddball paradigm to elicit mismatch negativity (MMN) in order to investigate the processing and representation of third tone sandhi words. This study used disyllabic /T2+T3/ (T2 condition), /T3+T4/ (T3 condition), and /T3+T3/ (sandhi condition) words as standards and an identical monosyllable [tʂu2] as the deviant in three separate conditions. The results in the first syllable time window showed that the T2 condition in which 竹叶 /tʂu2 yε4/ “bamboo” (T2) served as the standard and [tʂu2] as the deviant produced an MMN effect. They argued that this MMN effect was due to the surface acoustic differences between the first syllable of standards and the deviant. The results in the first syllable position for the T3 condition in which 主页 /tʂu3 yε4/ “main page” (T3) served as the standard and [tʂu2] as the deviant also elicited an MMN effect. This MMN effect could be due to the surface differences between the first syllable of standards and the deviant. It could also be due to differences in the underlying representation. Interestingly, no MMN effect was yielded in the first syllable position for the sandhi condition in which 主演 /tʂu3 jεn3/ “starring” served as the standard and [tʂu2] as the deviant. They argued that the results were probably because the participants perceived the deviant [tʂu2] as the first syllable of 主演 /tʂu3 jʂn3/ and converted the surface T2 into its underlying representation, or the representation of the first syllable of T3 sandhi words is phonologically underspecified, so there was no mismatch between the deviant and the first syllable of sandhi standards. According to their results, it seems that the surface acoustic information of T3 sandhi words is not that important when the experimental condition can help participants predict the following word. Retrieval of the underlying phonological representations is the key point.

In addition to on-line processing in the perception of Mandarin T3 sandhi words, there was one study working on on-line processing in the production of Mandarin T3 sandhi words. Zhang et al. (2015) investigated Event-Related Potentials (ERPs) in the covert production of Mandarin third tone sandhi in disyllabic words. Their stimuli included real words and pseudowords with T2-T3 and T3-T3 tonal combinations. The results showed that in comparison to the disyllabic words with T2-T3, the second syllable of the sandhi words with T3-T3 induced greater P2 (which is sensitive to phonological processing) amplitude. Zhang et al. (2015) claimed that the results suggest that the phonological encoding of tonal combinations with T3 sandhi may be more effortful. They further claimed that the phonological processing may not differ qualitatively between real words and pseudowords in the P2 time-window. In addition, the findings indicated that the phonetic/phonological encoding of T3 sandhi occurs before initiation of articulation. This research revealed on-line processing in the production of T3 sandhi words in Mandarin.

Previous studies on Mandarin T3 sandhi focused either on the acoustic and perceptual neutralization between SR-T3 and CR-T2 (Peng, 2000), or on how T3 sandhi words are processed and represented in the mental lexicon (e.g., Nixon et al., 2015; Chien et al., 2016, Chien et al., 2020). Few studies examined the dynamic processing between SR-T3 and CR-T2 as the acoustic signal of SR-T3 and CR-T2 unfolds. In this study, we not only revisited the extent of neutralization between SR-T3 and CR-T2 in both production and perception, but most importantly, also investigated the role of acoustic details within category in dynamic and automatic processing of tonal alternations in contexts. In order to approach this issue, we adopted an eye-movement tracking technique to provide detailed on-line processing information. It has shown in the previous eye-tracking studies that participants are aware of within-category differences in VOT (McMurray et al., 2002, 2009). In line with this, the current study employed the visual-word paradigm in eye-tracking, which taps into automatic processes, to investigate whether native listeners can perceive the differences between the two tones, at the suprasegmental level, and whether the acoustic details in lexical tone can facilitate lexical access in Mandarin. It is expected that the findings can shed light on the role of categorical perception and acoustic details in the processing of Mandarin tonal alternations in contexts. In addition, the current results can detect the subtle dynamic processing of disyllabic words with SR-T3 or CR-T2, as well as provide a hint for later stage of spoken word recognition. Specifically, the main research questions are as follows: (1) Are SR-T3 and CR-T2 acoustically incompletely neutralized? (2) Are SR-T3 and CR-T2 perceptually completely neutralized? (3) Are native Mandarin listeners sensitive to the acoustic details between SR-T3 and CR-T2 and able to use the information automatically in lexical access? The current study included three experiments. The first one was a speech production task which compared SR-T3 and CR-T2 to see whether we could replicate previous studies showing incomplete neutralization in F0 between them. The second one was an identification task and the last one was an eye-tracking experiment. The identification task tapped into phonological level since it induced more categorical processing, while the eye-tracking experiment tapped into automatic processing, on which level Mandarin listeners may show stronger sensitivity to subtle acoustic details.

## Experiment 1: Production

The speech production experiment aimed to replicate previous studies which observed incomplete neutralization in F0 between sandhi-rising tone 3 (SR-T3) and canonical-rising tone 2 (CR-T2) (Peng, 2000; Zhang and Lai, 2010; Yuan and Chen, 2014). This experiment also served as the ground for the critical stimuli used in Experiment 2 (identification) and Experiment 3 (eye-tracking). We predict that systematic differences in F0 between SR-T3 and CR-T2 would be obtained. Specifically, SR-T3 would show lower average F0, a larger F0 difference between the onset and turning point, and a later turning point than CR-T2, indicating the influence of their respective underlying representations.

### Participants

Twenty native Mandarin Chinese speakers from Northern China, aged between 20 and 24, were recruited (10 males and 10 females). None of them spoke any other Chinese dialects at the time of testing. They were also not simultaneous bilingual or early bilingual speakers of another non-Chinese language. All participants were university students with no reported language disability or hearing impairment. This research was reviewed and approved by the Human Subjects Committee of the Department of Chinese Language and Literature at Fudan University. All participants were asked to provide informed consent before the production experiment and were paid for their participation.

### Stimuli

Ten minimal pairs of disyllabic T3 (SR) + T3 and T2 (CR) + T3 words with identical segments were used as critical stimuli (e.g., 百马 /paj3 ma3/ “hundreds of horses” vs. 白马 /paj2 ma3/ “white horse”). These two sets of words were adopted from Zhang et al. (2015) and matched in log word frequency [SR-T3 words: *M* = 0.480, *SD* = 1.046; CR-T2 words: *M* = 0.899, *SD* = 1.088; *t*(18) = 0.876, *p* = 0.393] (based on the corpus of Cai and Brysbaert, 2010) and stroke number [SR-T3 words: *M* = 8.2, *SD* = 4.54; CR-T2 words: *M* = 8.5, *SD* = 3.60; *t*(18) = 0.164, *p* = 0.872]. In addition, their first morphemes can be combined with several other morphemes to form disyllabic words. Another twenty disyllabic words with different tonal combinations were also included as fillers. For the fillers, 8 of them started with T1; 4 of them started with T2; 8 of them began with T4. Eight of them ended with T1; 4 of them ended with T2; 8 of them ended with T4. Information of the 20 critical stimuli is shown in Appendix Table A1.

### Procedure

First, participants completed a language background questionnaire and a consent form in a quiet room. Then they did the production experiment run by Paradigm (Tagliaferri, 2019) and were recorded in the Phonetics and Psycholinguistics Lab at Fudan University, with a cardioid microphone (Shure, model SM57) and a digital solid-state recorder (Zoom H4N), using a sampling rate of 44,100 Hz.

In each trial of the production experiment, the participants first saw a fixation cross in the middle of the screen for 500 ms, and then the stimuli for 2,000 ms, during which they were instructed to produce the stimuli as naturally as possible. Five practice trials were first provided to the participants to ensure that the participants fully understood the procedure of the task. Then the main experiment began with a total of 120 tokens (20 critical stimuli and 20 fillers with three repetitions) randomly presented to the participants. The whole experiment took approximately 20 min. Participants’ productions of the critical stimuli were subjected to further analysis.

### Data Analysis

The F0 tracks of the first vowel of the SR-T3 and CR-T2 words were measured using Praat software (Boersma and Weenink, 2019) and defined from the onset of periodicity in the waveform to the peak of the pitch track analysis in Praat. F0 tracks were extracted using ProsodyPro Praat script (Xu, 2005/2010) and measured at every 11.11% of the F0 duration, generating 10 measurement points for each target vowel. Then the extracted F0 tracks were checked for octave jumps. Whenever there were octave jumps, the target vowel was equally divided into ten points and the value of each point was manually calculated using F0 = 1/T(s) in which T represents the duration of one period of the waveform. A total of 27 tokens (27/1,200 = 2.25%) were discarded due to creakiness (17 of them) or mispronunciation (10 of them). All tokens were judged by the two authors, who are native speakers of Mandarin Chinese.

The extracted F0 tracks using ProsodyPro were then converted into semi-tone using the formula in (1) below in order to better reflect pitch perception (Rietveld and Chen, 2006). Moreover, the semi-tone values were transformed into z-scores using the formula in (2) below to minimize variation due to gender and speaker identity (Rose, 1987; Zhu, 2004). The z-scores were subjected to statistical analysis.


(1) S⁢T=39.87×log⁢(H⁢z/50)

(2) $$z_{STx}=\frac{STx-\frac{1}{n}\sum_{i=1}^{n}\;STi}{\sqrt{\frac{1}{n-1}\sum_{i=1}^{n}(STi-\frac{1}{n}\sum_{i=1}^{n}\;STi){\;}^{2}}}$$

### Results and Discussion

**FIGURE 1:**
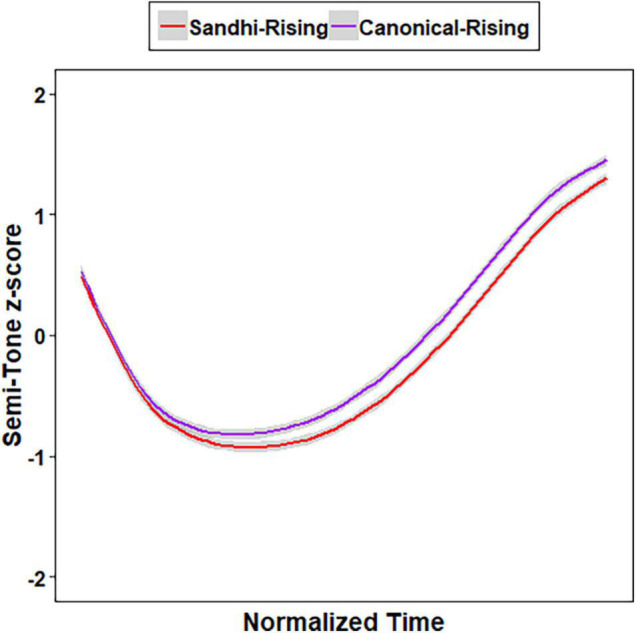
Mean semi-tone z-scores of the Sandhi-Rising T3 and Canonical-Rising T2 syllables.

Growth curve analysis (Mirman, 2014) were conducted to model the semi-tone z-scores represented by ten data points of SR-T3 and CR-T2 using the *lme4* package in R (Bates et al., 2015), with *p*-values calculated by the *lmerTest* package (Kuznetsova et al., 2017). The linear, quadratic, and cubic time polynomials were entered as fixed factors. Three models were created by adding the three time polynomials one at a time as fixed factors and as random slopes for the participant random effect. A series of likelihood ratio tests were conducted to compare between the three models. The model that could explain the most variance of the data was determined the best model. Results showed that the model consisting of all the three terms was the optimal [linear vs. linear + quadratic: χ^2^(4) = 7,307.1, *p* < 0.001; linear + quadratic vs. linear + quadratic + cubic: χ^2^(5) = 1,389.1, *p* < 0.001], within which all the three terms were significant, indicating that the F0 tracks of SR-T3 and CR-T2 had an incomplete S-shape on an angle, as shown in Figure 1 (linear: β = 1.547, *SE* = 0.090, *t* = 17.708, *p* < .001; quadratic: β = 1.840, *SE* = 0.069, *t* = 26.566, *p* < .001; cubic: β = –0.656, *SE* = 0.031, *t* = –20.769, *p* < .001).

In order to further evaluate whether or not canonical-rising T2 (CR-T2) and sandhi-rising T3 (SR-T3) were acoustically completely neutralized in tone, two additional models were built based on the best model above in which three time terms were included. Model A included all the three time polynomials and Tone (CR-T2, SR-T3, with CR-T2 serving as the baseline) as fixed factors. Model B included the three time polynomials, Tone, and their interactions as fixed factors. For both models, a set of random effects were also included to capture participant-level variability in all three time polynomials and in Tone. Results of likelihood ratio tests showed that Model B was significantly better than Model A [χ^2^(3) = 28.967, *p* < 0.001]. Within Model B, all three time polynomials were significant, indicating that the F0 tracks of SR-T3 and CR-T2 (the baseline) had an incomplete S-shape on an angle (linear: β = 1.622, *SE* = 0.089, *t* = 18.239, *p* < 0.001; quadratic: β = 1.795, *SE* = 0.071, *t* = 25.186, *p* < 0.001; cubic: β = –0.683, *SE* = 0.036, *t* = –19.021, *p* < 0.001). Moreover, SR-T3 showed significantly lower semi-tone z-scores than CR-T2, as reflected by the negative estimate for Tone (β = –0.141, *SE* = 0.039, *t* = –3.590, *p* = 0.002). Significant interaction effects between Tone and the linear time term (β = –0.151, *SE* = 0.034, *t* = –4.429, *p* < 0.001) as well as between Tone and the quadratic time term (β = 0.090, *SE* = 0.034, *t* = 2.637, *p* = 0.008) showed that the shapes of SR-T3 and CR-T2 were different. More specifically, the negative estimate for the interaction between the linear time term and Tone indicates that SR-T3 had a more negative slope compared to CR-T2, while the positive estimate for the interaction between the quadratic time term and Tone suggests that SR-T3 had a more convex shape (i.e., U shape) relative to CR-T2. The production results replicated those of previous studies (Peng, 2000; Zhang and Lai, 2010; Yuan and Chen, 2014), showing lower average F0 for SR-T3 and differences in F0 contour between the two tones. SR-T3 and CR-T2 were acoustically incompletely neutralized.

## Experiment 2: Identification

The identification experiment aimed to investigate whether native Mandarin listeners were perceptually sensitive to the acoustic differences between SR-T3, derived from third tone sandhi, and CR-T2. It examined native speakers’ categorical perception of Mandarin tonal alternations. In order for the identification stimuli to reflect the overall production pattern in F0, the SR-T3 tokens we used for the identification task had lower F0 than the CR-T2 tokens. Given the stimulus selection, better-than-chance signal detectability would suggest that native Mandarin listeners are sensitive to the subtle acoustic differences between the two tones, and able to use them for lexical access. Chance-level performance would suggest listeners’ inability to detect the tiny acoustic differences between SR-T3 and CR-T3 words during spoken word recognition.

### Participants

In the identification task, 32 native Mandarin listeners (18 females and 14 males; age range: 21–24 years old; mean age: 23.6 years old) from Northern China were recruited. None of them spoke any other Chinese dialects at the time of testing. They were also not simultaneous bilingual or early bilingual speakers of another non-Chinese language. None of them had participated in the production experiment. They were all university students with no reported language disability or hearing impairment. This research was reviewed and approved by the Human Subjects Committee of the Department of Chinese Language and Literature at Fudan University. All participants were asked to provide informed consent before the identification experiment and were paid for their participation.

### Stimuli

**FIGURE 2:**
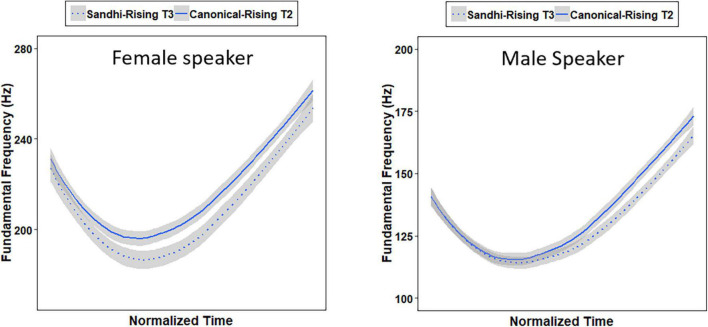
Mean F0 tracks of the Sandhi-Rising T3 and Canonical-Rising T2 syllables for the female (left panel) speaker and male (right panel) speaker.

The words used in the identification experiment were the same as the 10 pairs of critical words used in the production experiment (e.g., 百马 /paj3 ma3/ “hundreds of horses” vs. 白马 /paj2 ma3/ “white horse”) (Zhang et al., 2015). The auditory stimuli were taken from one Shandong female speaker and one Hebei male speaker’s productions in Experiment 1, whose average F0 of the 10 pairs of words was closely matched with that produced by all the 20 speakers. The selected SR-T3 tokens were always lower than the selected CR-T2 tokens in average F0 (see Figure 2), which was consistent with the statistical results obtained in the production experiment. In addition, the mean first syllable duration of the SR-T3 words was 272 ms, while the mean first syllable duration of the CR-T2 words was 260 ms. The mean second syllable duration for both groups of words was 411 ms. Independent-samples *t*-tests showed that neither the first syllable duration [*t*(38) = –1.088, *p* = 0.283] nor the disyllable duration [*t*(38) = –0.436, *p* = 0.665] was significantly different between the two groups.

### Procedure

First, participants completed a language background questionnaire and a consent form in the Phonetics and Psycholinguistics Lab at Fudan University. Then they did the forced-choice identification task run by Paradigm (Tagliaferri, 2019). During each trial, the participants first saw a fixation cross in the middle of the screen for 1,000 ms. As soon as it disappeared, a pair of SR-T3 and CR-T2 words with the tonal pattern of T3+T3 and T2+T3 were shown in Simplified Chinese characters (for example, 白马 vs. 百马) for 3,000 ms, and the participants were instructed to look at the two words during this time. The SR-T3 word always appeared to the right of the previously presented fixation cross and the CR-T2 word to the left. After the disappearance of the two words, the participants heard one of them via headphones. Immediately after the offset of the auditory stimulus, the two words were shown on the screen again. The participants were requested to identify which word they just heard by clicking the mouse, with the right button representing the SR-T3 word and the left button referring to the CR-T2 word. Before the main experiment, eight practice trials were presented to ensure that all the participants understood the experimental procedure. The 20 critical stimuli produced by the male speaker and the 20 by the female speaker were presented in two separate blocks. The block order was counterbalanced across participants and the trials were randomized within each block. The whole experiment took approximately 20 min.

### Results and Discussion

Mandarin listeners’ identification performance was evaluated using the formula in (3) for calculating A-prime scores (Grier, 1971; Snodgrass et al., 1985; Peng, 2000; So and Best, 2010), which reflect signal detectability and consider not only correct responses, but also false alarms. A’ scores range from 0 to 1, with a score of 1 indicating perfect performance and 0.5 representing random responses.

(3) $$A’=0.\text{5}+{\left[\left(y\;–\;x\right)(\text{1}+y\;–\;x)/\text{4}y\left(\text{1 }–\;x\right)\right]}^{\text{1}}$$^1^

The 32 Mandarin listeners’ mean A’ score was 0.517 with a standard deviation of 0.024. A one-sample *t*-test was conducted on participants’ A’ scores with a test value of 0.5. Although the mean A’ score was numerically very close from 0.5, it was still statistically significantly different from 0.5 [*t*(31) = 3.944, *p* < 0.001], suggesting that Mandarin listeners may be sensitive to the subtle acoustic differences between SR-T3 and CR-T2 in the forced-choice identification task, which required controlled processes.

In order to further understand the results of the A’ score, a series of generalized linear mixed-effects models were conducted on participants’ accuracy data using the lme4 package (Bates et al., 2015) in R, with *p*-values calculated using the lmerTest package (Kuznetsova et al., 2017). Participants’ accuracy was entered as a binomial dependent variable, with correct responses coded as 1 and incorrect responses coded as 0. Condition (SR-T3 vs. CR-T2), Talker (Female Talker vs. Male Talker), and their interactions were treated as fixed factors. For Condition, CR-T2 was set as the baseline to which SR-T3 was compared, while for Talker, Female Talker was regarded as the baseline to which Male Talker was compared. Participant and Item were entered as random factors. Likelihood ratio tests using forward stepwise selection were conducted to determine the best model. The model that contained the most fixed factors and fit significantly better than the one with one less variable was determined as the optimal model and reported below.

Table 1 shows the results of the accuracy data obtained in the identification task. As can be seen, the negative coefficient estimate for Condition indicates that Mandarin listeners made significantly more errors for SR-T3 words than for CR-T2 words. The negative coefficient estimate for Talker reveals that Mandarin listeners made significantly more errors when hearing the male speaker’s stimuli than when hearing the female speaker’s stimuli. Since the interaction between Condition and Talker was significant, two subsequent generalized linear mixed-effects models were conducted on participants’ accuracy data within Male and Female talkers, respectively, with Condition (SR-T3 vs. CR-T2) as a fixed factor, and Participant and Item as random factors. Results showed that Mandarin listeners made significantly more errors for SR-T3 words than for CR-T2 words when hearing the female speaker’s stimuli (β = -1.026, *SE* = 0.330, *t* = –3.105, *p* = 0.002), while they made similar numbers of errors for SR-T3 and CR-T2 words when hearing the male speaker’s stimuli (β = 0.152, *SE* = 0.309, *t* = 0.494, *p* = 0.621). The result pattern may be due to the fact that the acoustic difference between the SR-T3 and CR-T2 stimuli produced by the female speaker was larger than that between the SR-T3 and CR-T2 stimuli produced by the male speaker.

**TABLE 1:** Fixed effect estimates for the best model results of the accuracy data in identifying SR-T3 words and CR-T2 words.

	Estimate	*SE*	*Z*	*p*
Intercept	0.718	0.232	3.101	0.002**
Condition	–0.986	0.314	–3.139	0.002**
Talker	–0.628	0.172	–3.644	<0.001***
Condition × Talker	1.145	0.240	4.783	<0.001***

p ≤ 0.1, *p ≤ 0.05, **p ≤ 0.01, ***p ≤ 0.001.

**FIGURE 3:**
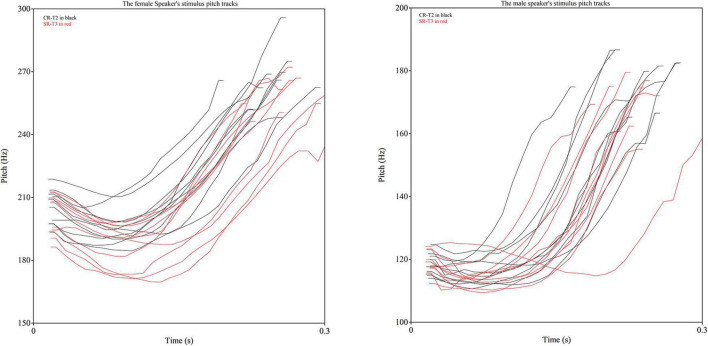
Raw F0 tracks of the Sandhi-Rising T3 and Canonical-Rising T2 syllables for the female (left panel) speaker and male (right panel) speaker.

These findings indicated that SR-T3 and CR-T2 may have demonstrated incomplete perceptual neutralization in identification, which is not consistent with previous studies showing complete neutralization in perception in the identification tasks (Wang and Li, 1967; Peng, 2000). The results may be due to the stimuli used in the current experiment, with SR-T3 words having a slightly lower average F0 value than CR-T2 words. To better understand the differences between studies, Figure 3 was created to capture the raw F0 tracks of individual stimuli produced by the female and male speakers. As displayed in Figure 3, there are considerable differences in the neutralization between speakers. The male speaker’s productions of SR-T3 and CR-T2 are almost fully overlapping in terms of the individual productions occupying in the same acoustic space, while the female speaker’s productions of the two tones include some productions from each tone group that occur outside the shared acoustic space, which may contain cues that the participants were picking up on during the identification experiment. The individual difference aspect of the speakers shown in Figure 3 is likely lead to differences between stimuli in different experiments, which may thus explain different findings in the perceptual neutralization between SR-T3 and CR-T2 across different studies.

Given that the forced-choice identification task required controlled processing and that the participants judged whether the target word was a T3+T3 (starting with SR-T3) or a T2+T3 (starting with CR-T2) word after hearing the whole disyllables, a question arose as to whether native Mandarin listeners would be able to recognize the target word (either a T3+T3 or a T2+T3 word) before hearing the whole disyllabic word. More specifically, it is worth examining the dynamic processing between SR-T3 and CR-T2 as the acoustic signal of SR-T3 and CR-T2 unfolds. It is also crucial to investigate the role of acoustic details within category in dynamic and automatic processing of the two incompletely neutralized tones in the sandhi context. In order to approach this issue, an eye-tracking experiment was conducted, which is argued to be very sensitive and implicit (McMurray et al., 2002, 2009; Qin et al., 2019), so that it allowed us to examine Mandarin listeners’ processing of SR-T3 (T3+T3) and CR-T2 (T2+T3) words before behavioral responses, such as word identification. The findings of the experiment would be able to shed light on the role of acoustic detail and phoneme/toneme during spoken word recognition.

## Experiment 3: Eye-Tracking

Since the A’ score in the identification experiment barely exceeded 0.5, we conducted an eye-tracking experiment with the visual world paradigm to further examine the extent of perceptual neutralization between SR-T3 and CR-T2. The eye-tracking technique has been utilized to show listeners’ sensitivity to within-category changes that are not usually captured by identification tasks which require participants’ overt responses (McMurray et al., 2002, 2009; Qin et al., 2019). Using such a method allows us to investigate whether Mandarin listeners are sensitive to the subtle acoustic differences between SR-T3 and CR-T2 during automatic processing stages as well as before overt behavioral responses. It also allows us to examine how SR-T3 and CR-T2 compete as the acoustic signal unfolds.

### Participants

In the eye-tracking experiment, 32 native Mandarin speakers (22 females and 10 males; age range: 20–28 years old; mean age: 23.7 years old) from Northern China were recruited. None of them spoke any other Chinese dialects at the time of testing. They were also not simultaneous bilingual or early bilingual speakers of another non-Chinese language. None of them had participated in Experiment 1 or Experiment 2. They were all university students with no reported language disability or hearing impairment. This research was reviewed and approved by the Human Subjects Committee of the Department of Chinese Language and Literature at Fudan University. All the participants were asked to provide informed consent before the eye-tracking experiment and were paid for their participation.

### Stimuli

The eye-tracking stimuli were the 40 disyllabic words used in Experiment 1, among which 10 were tone3 sandhi words (e.g., 百马 “hundreds of horses” /paj3 ma3/) and 10 were counterparts of the 10 tone3 sandhi words (e.g., 白马 “white horse” /paj2 ma3/). The two groups of critical stimuli were matched in log word frequency [SR-T3 words: *M* = 0.480, *SD* = 1.046; CR-T2 words: *M* = 0.899, *SD* = 1.088; *t*(18) = 0.876, *p* = 0.393] (based on the corpus of Cai and Brysbaert, 2010) and stroke number [SR-T3 words: *M* = 8.2, *SD* = 4.54; CR-T2 words: *M* = 8.5, *SD* = 3.60; *t*(18) = .164, *p* = .872] (see Appendix Table A1). In addition, their first morphemes can all be combined with several other morphemes to form disyllabic words. The remaining 20 disyllabic words were fillers with varied segments and tonal combinations. The auditory stimuli in the eye-tracking experiment were taken from the same Shandong female speaker and Hebei male speaker as Experiment 2, whose average F0 of SR-T3 and CR-T2 was closely matched with that produced by all the 20 speakers in Experiment 1. The disyllabic stimuli were presented in Simplified Chinese characters since not all of them were easily imageable (Huettig and McQueen, 2007; McQueen and Viebahn, 2007).

The 40 disyllabic words were further divided into 10 groups of four, with each group consisting of one tone3 sandhi word (SR-T3 word), the counterpart of the tone3 sandhi word (CR-T2 word), and two fillers. Within each group, every word served as the target once, and in every trial, the same four words appeared in a different location of an invisible 2 × 2 grid on the screen, resulting in a total of 40 trials. In addition, a given SR-T3 target was separated from its counterpart CR-T2 target by a minimum of ten trials, and vice versa. For example, the trial with 百马 (“hundreds of horses” /paj3 ma3/) as the target and the trial with 白马 (“white horse” /paj2 ma3/) as the target were at least ten trials apart. The location of targets was balanced across the 40 trials. Among the 40 trials, 20 of them had their targets produced by the male speaker, while the other 20 of them had their targets produced by the female speaker.

### Apparatus

Eye movements were recorded with an SR Research EyeLink 1000 Plus eye tracker at a sampling rate of 1,000 Hz. The visual stimuli were Simplified Chinese characters presented on a 19-inch LCD monitor with a resolution of 1,024 × 768 pixels using white text on a black background. The auditory stimuli were played by MIDIMAN M-TRACK 2X2M and Professional Monitor Headphones DJ-600 in order for accurate timing of sound presentation. The programming was performed using EyeLink Experiment Builder 2.1.140, and the eye-movement data were analyzed using EyeLink Data Viewer 3.1.97.

### Procedure

First, the participants completed a language background questionnaire and a consent form. Then they did the eye-tracking experiment in the Phonetics and Psycholinguistics Lab at Fudan University. The participants sat about 70 cm from the monitor with their head on a chin rest to reduce head movements. The experiment started with a 13-point calibration. Once this calibration check was completed accurately (<0.50 degrees of error), the experimenter advanced the screen to display four practice trials with feedback provided to participants, then followed by 40 trials of the main experiment without feedback. Within each trial, participants saw four disyllabic words presented for 5,000 ms, during which they were instructed to read the four words covertly in order to ensure that the phonological representations of the words were activated. Upon the disappearance of the four words, a fixation cross appeared in the middle of the screen for 500 ms during which participants were instructed to look at the fixation cross, so that their eye fixations would be brought to the display center. Immediately after the disappearance of the fixation cross, the four words reappeared on the screen in the same location with the sound of the target word simultaneously presented via headphones. Participants were requested to click on the target word with the mouse as quickly and accurately as possible upon hearing the target word. Participants’ eye fixations were measured from the onset of the auditory stimuli. Their behavioral responses were recorded as well. After the mouse click, the trial ended, with the next trial starting 2,000 ms later. The forty trials were equally separated into two blocks, with the targets in one block produced by the male speaker and the targets in the other block produced by the female speaker. Trials were randomly presented within each block, while the block order was counterbalanced across participants. The whole experiment consisted of 40 trials (10 SR-T3 words, 10 CR-T2 words, 20 fillers) and lasted approximately 20 min.

### Data Analysis

Participants’ eye movements in the four regions of interests corresponding to the four words on the screen were analyzed. Proportions of fixations to targets, competitors, and distractors were extracted with an 8-ms time window from the onset of the sound presentation to 1,256 ms after the onset, resulting in 157 bins. Ratios of proportions of fixations to targets over proportions of fixations to targets and competitors were calculated for the SR-T3 and CR-T2 target conditions, respectively, and named as target ratios; ratios of proportions of fixations to competitors over proportions of fixations to targets and competitors were generated for the SR-T3 and CR-T2 competitor conditions, individually, named as competitor ratios. Statistical analyses were conducted on SR-T3 target ratios and CR-T2 competitor ratios when SR-T3 words were the target (i.e., hearing SR-T3 words), on CR-T2 target ratios and SR-T3 competitor ratios when CR-T2 words were the target (i.e., hearing CR-T2 words), on the proportions of fixations to SR-T3 words when serving as both the target and competitor, and on the proportions of fixations to CR-T2 words when serving as both the target and competitor.

Four series of growth curve analyses (Magnuson et al., 2007; Mirman et al., 2008; Mirman, 2014; Connell et al., 2018) were conducted using the lme4 package (Bates et al., 2015) in R, with *p*-values calculated using the lmerTest package (Kuznetsova et al., 2017). Target ratios, competitor ratios, and participants’ proportions of fixations to CR-T2 and SR-T3 words between the 200 and 1,256 ms time window were modeled to accommodate the time that eye movements need to reflect speech processing (Hallett, 1986; Salverda et al., 2014). The end point of this time window was determined based on the duration of the 20 critical stimuli (around 675 ms) and participants’ reaction times in the identification task of Experiment 2 (around 1,150 ms).

For the first series of analyses, SR-T3 target ratios and CR-T2 competitor ratios were modeled; for the second series, CR-T2 target ratios and SR-T3 competitor ratios were modeled; for the third series, SR-T3 words served both as the target and competitor; for the fourth series, CR-T2 words served both as the target and competitor. All series included Condition as a fixed factor (two levels). For Condition, CR-T2 was treated as the baseline to which SR-T3 was compared for the first two series of analyses. For the third series, SR-T3 competitor was treated as the baseline to which SR-T3 target was compared, while for the fourth series, CR-T2 target was deemed the baseline to which CR-T2 competitor was compared. Time (linear, quadratic, cubic) and interactions between Time and Condition were also included as fixed factors to capture the non-linear nature of the eye-tracking data. In addition, all analyses also included a set of random effects to capture Participant-level and Participant-by-Condition variability in the three time polynomials (Mirman, 2014). Likelihood ratio tests using forward stepwise selection were conducted to determine the best model for all series of analyses. The model that contained the most fixed factors and fit significantly better than the one with one less variable was determined as the optimal model and reported below.

The former two series of analyses allow us to investigate how acoustically incompletely neutralized SR-T3 and CR-T2 words compete as the acoustic signal unfolds within the same trial. The latter two serious of analyses grant us the opportunity to compare the recognition process between identical visual stimuli when serving as the target and competitor in different trials. If participants look more to the target words relative to their corresponding competitors, and if proportions of fixations to targets increase more strongly as a function of time, results would further support those in Experiment 2, indicating that Mandarin listeners are sensitive to the subtle acoustic details between SR-T3 and CR-T2. Therefore, Mandarin listeners should not only show significant differences between target and competitor ratios, between proportions of fixations to SR-T3 targets and competitors, and between proportions of fixations to CR-T2 targets and competitors, but also reveal significant interactions between Condition and at least one of the time polynomials (linear, quadratic, cubic). Such interactions would suggest that the difference between target and competitor ratios changed significantly during the course of target recognition, and so did the difference between the proportions of fixations to the SR-T3 target and competitor, and between the proportions of fixations to the CR-T2 target and competitor.

### Results and Discussion

**FIGURE 4:**
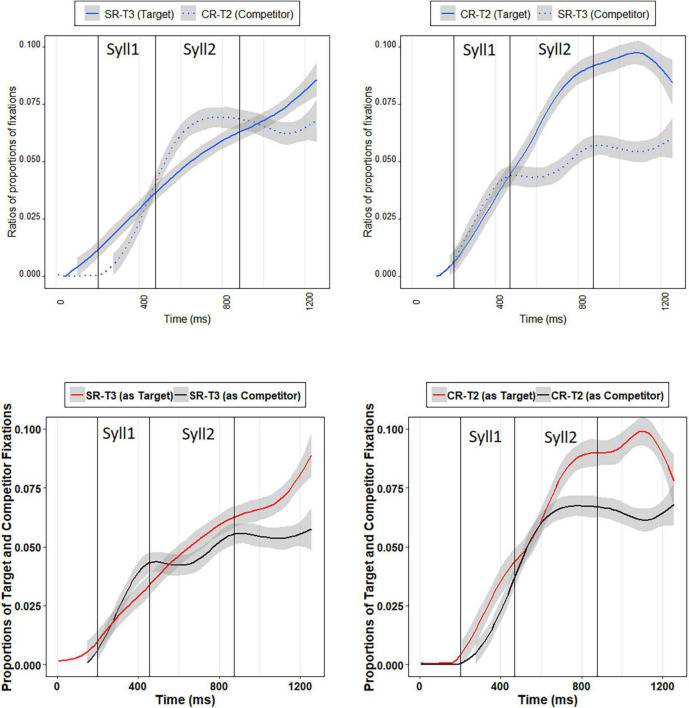
Top-left panel: SR-T3 target ratios and CR-T2 competitor ratios when Mandarin listeners hearing SR-T3 words; top-right panel: CR-T2 target ratios and SR-T3 competitor ratios when Mandarin listeners hearing CR-T2 words; bottom-left panel: Mandarin listeners’ proportions of fixations to SR-T3 targets and SR-T3 competitors; bottom-right panel: Mandarin listeners’ proportions of fixations to CR-T2 targets and CR-T2 competitors.

Table 2 presents the results of the growth curve analysis with the best fit on the ratios of SR-T3 targets (e.g., 百马 “hundreds of horses” /paj3 ma3/) and CR-T2 competitors (e.g., 白马 “white horse” /paj2 ma3/) when hearing SR-T3 words. As summarized in Table 2, the positive estimate for the interaction between the quadratic time polynomial and Condition indicated that the competitor ratio curve is more of a concave shape (i.e., upside-down U shape) than the target ratio curve. Interestingly, the effect of Condition was not significant, indicating that Mandarin listeners did not look more to the SR-T3 words compared to the CR-T2 words when hearing the SR-T3 words in the 200–1,256 ms time window. The lack of the effect of Condition may be due to the fact that Mandarin listeners looked more to SR-T3 words (tone 3 sandhi words) in the first syllable time window. However, they reconsidered CR-T2 words after hearing the second syllable. Shortly after the end of the second syllable, they looked more to the SR-T3 words again (see Figure 4), which were the targets. The crossing of the SR-T3 and CR-T2 fixation curves may have led to the insignificance of Condition.

**TABLE 2:** Results of growth curve analysis on SR-T3 target ratios and CR-T2 competitor ratios when hearing SR-T3 words.

	Estimate	*SE*	*t*	*p*
Intercept	0.049	0.019	2.521	0.017*
**Time**				
Linear	0.190	0.081	2.362	0.024*
Quadratic	–0.156	0.060	–2.618	0.011*
Cubic	0.023	0.021	1.091	0.280
Condition	0.001	0.006	0.139	0.890
**Interaction**				
Linear × Condition	0.018	0.038	0.459	0.649
Quadratic × Condition	0.123	0.066	1.852	0.068.
Cubic × Condition	–0.009	0.026	–0.351	0.727

p ≤ 0.1, *p ≤ 0.05, **p ≤ 0.01, ***p ≤ 0.001.

Table 3 demonstrates the results of the growth curve analysis with the best fit on the ratios of CR-T2 targets (e.g., 白马 “white horse” /paj2 ma3/) and SR-T3 competitors (e.g., 百马 “hundreds of horses” /paj3 ma3/) when hearing CR-T2 words. The negative estimate for the effect of Condition indicates that Mandarin listeners looked more to the CR-T2 words than to the SR-T3 words in the 200–1,256 ms time window when the CR-T2 words were the target. The negative estimate for the interaction between the linear time polynomial and Condition suggests that the target ratio curve has a more positive slope than the competitor ratio curve. As Figure 4 shows, Mandarin listeners could not distinguish between the CR-T2 words and SR-T3 words in the first syllable time window, as evidenced by the two adjacent ratio curves before the offset of the first syllable. However, after the onset of the second syllable, they started to look more to the CR-T2 words (target). The CR-T2 advantage persisted into the post-lexical time window.

**TABLE 3:** Results of growth curve analysis on CR-T2 target ratios and SR-T3 competitor ratios when hearing CR-T2 words.

	Estimate	*SE*	*t*	*P*
Intercept	0.066	0.022	2.982	0.005**
**Time**				
Linear	0.298	0.099	3.002	0.004**
Quadratic	–0.123	0.051	–2.423	0.019*
Cubic	–0.026	0.046	–0.572	0.569
Condition	–0.022	0.010	–2.123	0.041*
**Interaction**				
Linear × Condition	–0.182	0.103	–1.778	0.082.
Quadratic × Condition	0.064	0.055	1.154	0.253
Cubic × Condition	0.053	0.062	0.861	0.392

p ≤ 0.1, *p ≤ 0.05, **p ≤ 0.01, ***p ≤ 0.001.

Table 4 shows the results of the growth curve analysis with the best fit on the proportions of fixations to SR-T3 words (e.g., 百马 “hundreds of horses” /paj3 ma3/) when serving as the target and competitor. As can be seen in Table 3, neither Condition nor any of the time polynomial and Condition interactions were significant, indicating that Mandarin listeners’ overall proportions of fixations to the SR-T3 targets were not different from those to the SR-T3 competitors in the 200–1,256 ms time window, neither did they change distinctively as a function of time (see Figure 4). The lack of significance in Condition may be due to the fact that Mandarin listeners did not look more to SR-T3 targets until the post-lexical time window, which is consistent with the situation in which the ratios of SR-T3 targets were compared with those of CR-T2 competitors when SR-T3 words were the target.

**TABLE 4:** Results of growth curve analysis on Mandarin listeners’ proportions of fixations to SR-T3 targets and SR-T3 competitors.

	Estimate	*SE*	*t*	*P*
Intercept	0.043	0.018	2.355	0.025*
**Time**				
Linear	0.115	0.078	1.484	0.144
Quadratic	-0.059	0.037	–1.600	0.116
Cubic	–0.025	0.035	0.743	0.461
Condition	0.006	0.006	0.906	0.372
**Interaction**				
Linear × Condition	0.090	0.077	1.171	0.248
Quadratic × Condition	0.026	0.038	0.681	0.500
Cubic × Condition	–0.013	0.043	–0.299	0.767

p ≤ 0.1, *p ≤ 0.05, **p ≤ 0.01, ***p ≤ 0.001.

Table 5 displays the results of the growth curve analysis with the best fit on the proportions of fixations to CR-T2 words (e.g., 白马 “white horse” /paj2 ma3/) when serving as the target and competitor. The negative estimate for the effect of Condition indicates that Mandarin listeners’ overall proportions of fixations to the CR-T2 targets were higher than those to the CR-T2 competitors in the 200–1,256 ms time window. The negative estimate for the interaction between the linear time polynomial and Condition indicates that the CR-T2 targets’ fixation curve has a more positive slope than the CR-T2 competitors’ fixation curve. As Figure 4 reveals, Mandarin listeners did not look more to the CR-T2 targets before the first half of the second syllable. After the middle of the second syllable, they started to look more to the CR-T2 targets. This pattern persisted into the post-lexical time window. These results were in line with those in which ratios of CR-T2 targets were compared with those of SR-T3 competitors when CR-T2 words served as the target.

**TABLE 5:** Results of growth curve analysis on Mandarin listeners’ proportions of fixations to CR-T2 targets and CR-T2 competitors.

	Estimate	*SE*	*t*	*P*
Intercept	0.065	0.023	2.904	0.006**
**Time**				
Linear	0.296	0.101	2.941	0.006
Quadratic	–0.123	0.068	–1.814	0.077.
Cubic	–0.025	0.036	–0.698	0.488
Condition	–0.017	0.008	–2.178	0.034*
**Interaction**				
Linear × Condition	–0.106	0.055	–1.930	0.062.
Quadratic × Condition	–0.027	0.042	–0.637	0.528
Cubic × Condition	0.047	0.040	1.162	0.254

p ≤ 0.1, *p ≤ 0.05, **p ≤ 0.01, ***p ≤ 0.001.

Taken together, these eye-tracking results seem to suggest that Mandarin listeners, in general, were able to differentiate SR-T3 words from CR-T2 words in the automatic processing stages. The results also suggest that SR-T3 was a more ambiguous tone, which confused Mandarin listeners before the sandhi context was fully revealed. Only shortly after the offset of the second syllable could Mandarin listeners utilize the subtle acoustic differences between SR-T3 and CR-T2 to recognize the target words. By contrast, CR-T2 did not exhibit such ambiguity, allowing Mandarin listeners to differentiate CR-T2 from SR-T3 words no later than the middle of the second syllable, indicating that immediately after the appearance of the sandhi context, Mandarin listeners could incorporate the contextual information of sandhi into the word recognition process.

In addition to the eye-tracking data, we also analyzed Mandarin listeners’ identification performance in the visual-world paradigm. As in Experiment 2, A-prime scores were calculated in order to evaluate listeners’ sensitivity between SR-T3 and CR-T2 words (Peng, 2000). Results showed that the mean A’ score was 0.511 with a standard deviation of 0.017. A one sample *t*-test was conducted to examine whether listeners’ A’ scores were significantly better than the chance level of 0.5. Consistent with the results of Experiment 2, the Mandarin listeners’ mean A’ score obtained in the word identification task of the visual-world paradigm was significantly better than chance [*t*(31) = 3.629, *p* = 0.001]. The eye-tracking results and the identification results obtained in the visual-world paradigm and in Experiment 2 together indicate that Mandarin listeners may be able to detect the subtle acoustic differences between the SR-T3 and CR-T2 words at automatic processing stages. This sensitivity then carries over into later processing stages to aid word recognition.

## General Discussion

The study revisits the issue of perceptual neutralization between SR-T3 and CR-T2 in the literature. Based upon previous research, the current study employs the eye-tracking technique, which can provide on-line processing data, to examine tone sandhi in the context of disyllabic words. In order to investigate the extent of neutralization between SR-T3 and CR-T2, this study conducts three experiments, including the production experiment, the identification experiment, and the eye-tracking experiment, to integrate the findings from acoustic analysis, perceptual recognition, and cognitive processing.

For the production data, the acoustic analysis demonstrates that SR-T3 and CR-T2 are different in F0 contour and SR-T3 has lower average F0 than CR-T2. The results replicate those of previous studies (Peng, 2000; Zhang and Lai, 2010; Yuan and Chen, 2014). Thus, the findings in the production analysis suggest that SR-T3 and CR-T2 are acoustically incompletely neutralized.

From the identification task, the results show that Mandarin listeners tend to be aware of the subtle acoustic differences between SR-T3 and CR-T2 in the forced-choice perception task, as indicated that SR-T3 and CR-T2 are perceptually different. The current results are not consistent with previous research by Peng (2000), which showed Mandarin listeners failed to correctly categorize these two tones. It is probably because the stimuli used in the current study can reflect actual production patterns of the two tones; that is, SR-T3 has generally lower average F0 than CR-T2. It is also likely due to individual differences in the productions of the two tones between different speakers across studies. Despite these potential differences, the current findings in the identification task suggest that SR-T3 and CR-T2 are perceptually incompletely neutralized.

In the eye-tracking results, we compared target ratios and competitor ratios when hearing SR-T3 and CR-T2 words, respectively. When hearing SR-T3 words, Mandarin listeners looked more to SR-T3 in the first syllable time window. After encountering the sandhi context (i.e., hearing the second syllable), however, they started to consider the target tone as CR-T2 and looked more to CR-T2 words in the second syllable time window. During the entire word window, they looked to both SR-T3 and CR-T2 words. Then, shortly after the end of the entire words, they looked more back to SR-T3 words in the post-lexical time window. We speculate that SR-T3 (high-rising tone with a lower average pitch) is marked so it first drew Mandarin listeners’ attention, whereas CR-T2 is in the tonal inventory. Thus, it did not stand out until the listeners heard the onset of the second syllable and reconsidered CR-T2 words. When hearing CR-T2 words, Mandarin listeners looked more to CR-T2 words than to SR-T3 words overall, but they were not sensitive to the differences between the two tones and could not distinguish the two tones in the first syllable time window. Immediately after they encountered the sandhi context (i.e., upon hearing the second syllable), Mandarin listeners looked more to the CR-T2 words toward the end of entire words.

We also compared the proportion of fixations to SR-T3 targets with that of SR-T3 competitors as well as compared the proportion of fixations to CR-T2 targets with that of CR-T2 competitors. The results suggest that SR-T3 was a more ambiguous tone. When hearing SR-T3 words, Mandarin listeners tended to be confused between SR-T3 and CR-T2 until the sandhi context was fully revealed, as shown by the fact that the fixation curve of SR-T3 targets was not significantly different from that of SR-T3 competitors (i.e., when hearing CR-T2 words), indicating that both CR-T2 and SR-T3 words were activated to a similar degree until the sandhi context was fully revealed. By contrast, the results seem to show a bias toward CR-T2 words in the sense that even when hearing SR-T3 words, the proportion of fixations to CR-T2 words (CR-T2 competitors) was not different from that to CR-T2 targets until the middle of the second syllable. These results are probably because CR-T2 is in the tonal inventory while SR-T3 is not, and therefore SR-T3 is more ambiguous than CR-T2.

The current results support that perception of tone is an incremental process in that the pitch height at critical points serves as an important perceptual cue. The sandhi context, i.e., the appearance of the second T3 syllable, is at play for identifying SR-T3 or CR-T2 in early processing stages of spoken word recognition. In sum, the findings demonstrate that Mandarin listeners tend to process CR-T2 as T2 whereas they tend to first process SR-T3 as both T3 and T2, and later detect the acoustic differences between the two tones revealed by the sandhi context, and finally activate the target word during lexical access. The findings in the eye-tracking experiment suggest that Mandarin listeners are sensitive to the acoustic details between SR-T3 and CR-T2 and able to use the information automatically in lexical access.

## Concluding Remarks

This study explores the extent of neutralization of SR-T3 and CR-T2 in Mandarin. Mandarin T3 sandhi is traditionally/pedagogically described as tonal neutralization within category; that is, a T3 is altered to a T2 when it is followed by another T3. The results in previous studies showed inconsistencies in that SR-T3 and CR-T2 were incompletely neutralized in acoustic details but completely neutralized in perceptual identification. The current study aims to resolve those inconsistencies by conducting the production, perception, and eye-tracking experiments. The production and perception results show that SR-T3 and CR-T2 are incompletely neutralized in acoustics and perception. In addition, the eye-tracking results show that native Mandarin listeners can distinguish the differences between SR-T3 and CR-T2. The eye-tracking data further demonstrate the on-line processing of tonal alternations in sandhi contexts; that is, Mandarin listeners tend to perceive SR-T3 as SR-T3 and CR-T2 for the entire word window, whereas they tend to process CR-T2 as both tones only in the first syllable position, then detect the acoustic differences between the two tones revealed by the sandhi context, and eventually retrieve the target word. In conclusion, our findings suggest that native Mandarin listeners are able to use not only the detailed acoustic differences “within category” in lexical access, but they also rely on phonological contexts to perceive phonetic differences. If purely acoustic-phonetic details could determine processing, then the listeners were supposed to be able to distinguish SR-T3 from CR-T2 words in the first syllable. The eye-tracking results, however, showed that the listeners generally could detect CR-T2 until the appearance of second syllable and SR-T3 until the post-lexical time window. In line with this, the results shed light on the hybrid model of lexical representation that considers both surface acoustic-phonetic information and the underlying representation during spoken word recognition (e.g., Deelman and Connie, 2001; Connine, 2004; Connine and Pinnow, 2006; Ranbom and Connine, 2007). Future studies should be conducted to examine what acoustic cues can be used by Mandarin listeners to help disambiguate between SR-T3 and CR-T2 during the recognition process. The present results also imply that it would be better to learn phonetic contrasts through vocabulary at the lexical level in language learning or training for language disorders.

## Data Availability Statement

The raw data supporting the conclusions of this article will be made available by the authors, without undue reservation.

## Ethics Statement

The studies involving human participants were reviewed and approved by the Zhongmin Chen, Department of Chinese Language and Literature, Fudan University Yueling Ping, Department of Chinese Language and Literature, Fudan University Liang Ma, Department of Chinese Language and Literature, Fudan University. The patients/participants provided their written informed consent to participate in this study.

## Author Contributions

J-YT contributed 60% of the work. Y-FC contributed 40% of the work. Both authors contributed to the article and approved the submitted version.

## Conflict of Interest

The authors declare that the research was conducted in the absence of any commercial or financial relationships that could be construed as a potential conflict of interest.

## Publisher’s Note

All claims expressed in this article are solely those of the authors and do not necessarily represent those of their affiliated organizations, or those of the publisher, the editors and the reviewers. Any product that may be evaluated in this article, or claim that may be made by its manufacturer, is not guaranteed or endorsed by the publisher.

## Footnotes

1 x is the ratio of false alarms (i.e., identification of SR-T3 words for CR-T2 words and that of CR-T2 words for SR-T3 words), and y is the ratio of correct responses (i.e., identification of SR-T3 words for SR-T2 words and that of CR-T3 words for CR-T3 words).

## Appendix

**TABLE A1:** The critical stimuli.

No.	Word	Tone		Log freq.	First syllable stroke number	Word	Tone		Log freq.	First syllable stroke number
1	儿语	er2	yu3	0	2	耳语	er3	yu3	1.792	6
2	淘米	tao2	mi3	0	11	讨米	tao3	mi3	0	5
3	白马	bai2	ma3	1.672	5	百马	bai3	ma3	0	6
4	白米	bai2	mi3	0.477	5	百米	bai3	mi3	0	6
5	毒瘾	du2	yin3	2.308	8	赌瘾	du3	yin3	0	12
6	财礼	cai2	li3	0	7	彩礼	cai3	li3	0.01	11
7	骑马	qi2	ma3	2.519	11	起码	qi3	ma3	2.991	10
8	黏米	nian2	mi3	0	17	碾米	nian3	mi3	0	15
9	无礼	wu2	li3	2.009	4	五里	wu3	li3	0	4
10	遗老	yi2	lao3	0	12	倚老	yi3	lao3	0.01	10
